# Myo-inositol moderates maternal BMI and glycemia related variations in in-vitro placental ^13^C-DHA-metabolism, altering their relationships with birthweight

**DOI:** 10.1038/s41598-022-18309-2

**Published:** 2022-09-01

**Authors:** Oliver C. Watkins, Preben Selvam, Reshma Appukuttan Pillai, Victoria K. B. Cracknell-Hazra, Hannah E. J. Yong, Neha Sharma, Amaury Cazenave-Gassiot, Anne K. Bendt, Keith M. Godfrey, Rohan M. Lewis, Markus R. Wenk, Shiao-Yng Chan

**Affiliations:** 1 Department of Obstetrics and Gynaecology, Yong Loo Lin School of Medicine, National University of Singapore, National University Health System, 1E Kent Ridge Road, NUHS Tower Block, Level 12, Singapore, 119228 Singapore; 2 Singapore Institute for Clinical Sciences, Agency for Science, Technology and Research, Singapore, Singapore; 3 Department of Biochemistry, Yong Loo Lin School of Medicine, National University of Singapore, Singapore, Singapore; 4 Singapore Lipidomics Incubator, Life Sciences Institute, National University of Singapore, Singapore, Singapore; 5 Faculty of Medicine, University of Southampton, Southampton, UK; 6 MRC Lifecourse Epidemiology Unit and NIHR Southampton Biomedical Research Centre, University of Southampton and University Hospital Southampton NHS Foundation Trust, Southampton, UK

**Keywords:** Lipidomics, Biochemistry, Developmental biology, Medical research, Molecular medicine

## Abstract

Transplacental docosahexaenoic-acid (DHA) supply for fetal development is regulated by placental DHA-lipid metabolism. Both maternal diabetes and obesity are linked to possible decreased fetal circulating DHA and increased placental DHA-lipids. Since myo-inositol is a promising intervention for gestational diabetes (GDM), we aimed to determine whether myo-inositol could rectify perturbations in placental DHA metabolism associated with maternal increasing glycemia and obesity and examine links with birthweight. Term placental villous explants from 17 women representing a range of BMIs and mid-gestational glycemia, were incubated with ^13^C-labeled-DHA for 48 h, in 0.3 µmol/L (control) or 60 µmol/L myo-inositol. Individual newly synthesized ^13^C-DHA-labeled lipid species were quantified by liquid-chromatography-mass-spectrometry. Compared with controls, incubation with myo-inositol decreased most ^13^C-DHA-lipids in placental explants from women with higher BMI or higher glycemia, but increased ^13^C-DHA-lipids with normal BMI or lower glycemia. Myo-inositol also increased ^13^C-DHA-labeled lipids in cases of lower birthweight centile, but induced decreases at higher centiles. Myo-inositol therefore lowered DHA-lipids in placenta with high basal placental DHA-lipid production (higher BMI and glycemia) but increased DHA-lipids where basal processing capacity is low. Myo-inositol thus moderates placental DHA metabolism towards a physiological mean which may in turn moderate birthweight.

## Introduction

During healthy pregnancy the placenta is able to regulate and preferentially transfer DHA from the maternal to fetal circulation^1^, with increased bio-magnification of DHA when maternal supply is low, ensuring optimal DHA supply^2^. However, this process is dysregulated in both maternal obesity and diabetes^1^,with suggestion of increased retention of DHA within the placenta and reduced supply to the fetus^3–12^. DHA and DHA-containing lipids are vital for fetal health and development, particularly brain growth^13,14^, but the fetus is reliant on maternal DHA supply due to a limited ability to synthesize DHA^13–15^. Placental DHA metabolism and fetal DHA-lipid supply have also been linked with birthweight^12,16–20^.

### Maternal metabolic characteristics and fetal growth

Both extremes of fetal growth are associated with obstetric complications and risk of neonatal morbidity and perinatal death^21^, as well as an increased long-term risk of cardiometabolic disorders including diabetes, obesity and cardiovascular disease^21–23^. Current practice aims at reducing fetal overgrowth in pre-existing maternal diabetes and gestational diabetes (GDM) by normalizing maternal glycemia. However, fetal macrosomia may occur in diabetes even when glycemia is well controlled, and also commonly occurs in obese mothers without diabetes^24–26^. This has led to the postulation that dysregulated transplacental lipid supply may also underlie fetal macrosomia and hyper-adiposity in these conditions, the specifics of which remain poorly defined^27^. At the other extreme, intrauterine growth restriction is commonly due to underlying uteroplacental insufficiency for which there is no proven treatment to restore fetal growth once growth restriction is evident. In addition to glucose and amino acids, lipids are a major class of macronutrients required for fetal growth^27^. There is thus an urgent need to understand the role of placental lipid metabolism in the pathogenesis of disordered fetal growth and to develop new strategies that can potentially address it.

### Myo-inositol, metabolism and adiposity

Myo-inositol is a carbohydrate with potential as a dietary intervention for pregnancies complicated by diabetes and obesity. Myo-inositol is the most abundant of nine inositol stereoisomers and is an important regulatory polyol, synthesized endogenously in humans (4 g daily by kidneys^28^) and obtained in diet from fruits, grains and nuts, with a typical Western diet providing about 1 g of inositol a day^29^. Inositol and inositol derivatives have important roles in lipid and glucose metabolism, including as insulin mimics and second messengers in signaling pathways^30,31^. Dysregulated inositol biology is associated with pregnancy complications, such as gestational diabetes and pre-eclampsia^31,32^, with reduced placental inositol in GDM postulated to be permissive to glycemia-induced acceleration of fetal growth^33,34^. Myo-inositol is currently being trialed as a potential treatment and preventive agent for GDM but there is little understanding of how it affects placental function^31^. In non-pregnant animal models, myo-inositol treatment altered body wide lipid distribution between different organs^31,35–38^, but effects were strongly dependent on body weight. For example, myo-inositol deficiency in normal weight rats increased adipose tissue lipid mobilization and increased hepatic lipid accumulation, while myo-inositol supplementation reversed these effects^31,35–37^. In contrast, myo-inositol supplementation of a high fat diet obese adult mouse model reduced fat accretion in white adipose tissue^31,38^. The effects of myo-inositol also appear to depend on BMI in humans. For example, myo-inositol supplementation in polycystic ovary syndrome (PCOS) decreased BMI in obese women^39,40^, but not in non-obese women^41–44^.

### Placental DHA metabolism

Placental DHA metabolism is central to the regulation of transplacental DHA supply to the fetus, as well as to local regulation of diverse aspects of placental function, including nutrient transport, intracellular and endocrine signaling, control of oxidative stress and others that regulate growth^16,24,27^. Our previous study, which measured the conversion of ^13^C-DHA into three individual ^13^C-DHA-triglycerides (TGs) in placental explants from mainly overweight non-GDM women, showed that myo-inositol treatment decreased ^13^C-DHA TG production^45^. We have since developed a new method that can measure an additional fourteen ^13^C-DHA-lipids and demonstrated that both maternal BMI and glycemia leave a lasting placental-effect that is detectable in placental culture and these maternal characteristics were positively associated with placental DHA-lipid producing capacity^12^.

### Hypothesis and aims

Placental DHA metabolism plays an important role in ensuring a healthy progress of pregnancy including fetal growth and development, but is dysregulated with both maternal obesity and dysglycemia^8,31,46^. Given the ability of myo-inositol to influence general lipid metabolism^31^, we examined if myo-inositol treatment in-vitro could rectify perturbations in the metabolism of DHA lipids in placental explants from women across a range of BMI and gestational glycemia. We also aimed to investigate if myo-inositol-induced alterations in placental DHA metabolism could be related to variations in birthweight. We hypothesized that myo-inositol treatment would optimize placental ^13^C-labeled DHA metabolism in cases of higher maternal glycemia or BMI, and that myo-inositol-induced changes would be associated with the moderation of birthweight.

## Results

Incubation of placental explants with stable-isotope ^13^C-DHA for 48 h resulted in production of stable-isotope labeled ^13^C-DHA-lipids. Seventeen could be quantified reliably with our targeted triple quadrupole LCMS method. Quantified ^13^C-DHA-lipids included two phosphatidyl-ethanolamine plasmalogens (PE-P 38:6, PE-P 40:6), phosphatidyl-choline (PC-38:6), lyso-phosphatidyl-choline (LPC 22:6), lyso-phosphatidyl-ethanolamine (LPE 22:6), three diacylglycerols (DG) and nine triacylglycerols (TG). Further description of ^13^C-DHA lipid abundance under control conditions can be found in our previous publication^12^.

### Myo-inositol treatment and change in amount of placental ^13^C-DHA lipids

As a group, placental explants treated with 60 µmol/L myo-inositol, showed no significant change in the mean amount of each placental ^13^C-DHA-lipid relative to the corresponding control cultured with no additional myo-inositol. There was wide inter-placental variation in effects, which we had hypothesized may be due to variance in programming by maternal BMI and glycemia. Hence, we next sought to associate each placental ^13^C-DHA-lipid’s myo-inositol response with these maternal factors.

### Association of maternal BMI with change in placental ^13^C-DHA lipids in response to myo-inositol treatment

**Figure 1:**
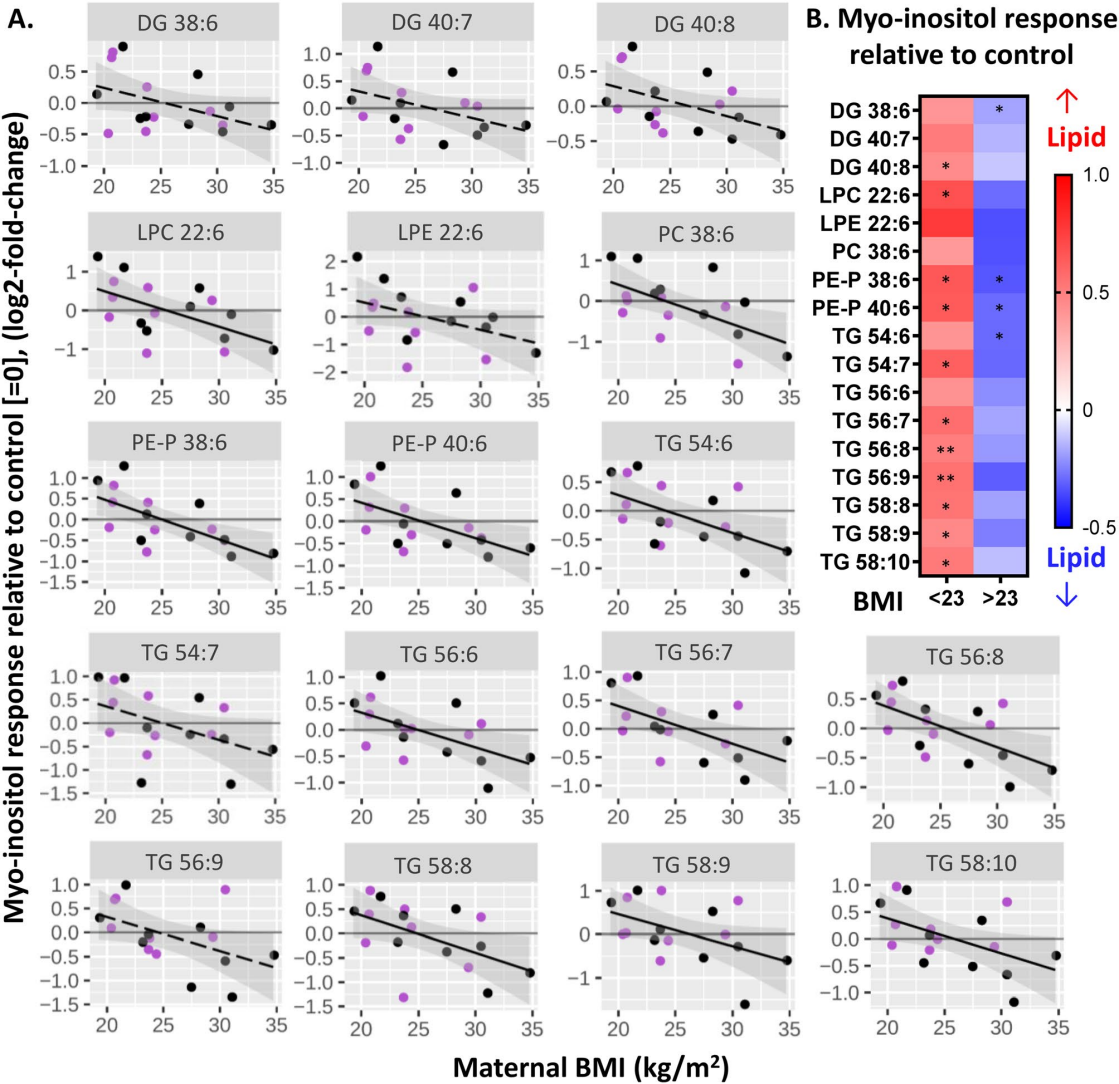
Associations between maternal BMI and ^13^C-DHA lipids in response to myo-inositol treatment (myo-inositol response). Myo-inositol response represents the relative amount of ^13^C-DHA lipid in placental explants treated with myo-inositol (60 µmol/L) compared with control explants from the same placenta. Positive values for myo-inositol response (log2-fold-change) indicate an increase in ^13^C-DHA lipids compared with the control (= 0), whilst negative values indicate a decrease. Linear regression was run with myo-inositol response as the outcome and BMI as the exposure/predictor variable (**A**). Solid lines show statistically significant associations while dashed lines show non-significant trends. Shaded areas show 95% confidence intervals. Purple: non GDM, Black: GDM. (**B**) Heat map illustrating myo-inositol response in placental explants from normal weight women (BMI < 23 kg/m^2^) and from overweight and obese women (BMI ≥ 23 kg/m^2^). Positive values (red) indicate an increase in ^13^C-lipids while negative values (blue) indicate a decrease. Asterisks indicate significant differences between 60 µmol/L myo-inositol treatment and controls from the same placenta not treated with myo-inositol. *p < 0.05, **p < 0.01. Benjamini–Hochberg was used to correct for multiple comparisons in all analyses.

Following on from our previous report that basal placental production of ^13^C-DHA-lipids increases with higher maternal BMI^12^, we have now investigated the alterations in response to myo-inositol treatment in-vitro. In relation to maternal BMI treated as a continuum, there was a negative association between BMI and placental ^13^C-DHA-lipids’ myo-inositol response, which was significant for ^13^C-DHA LPC, PC-38:6, PE-P 38:6, PE-P 40:6 and most ^13^C-DHA-TGs (Fig. 1, Table 1). Placental explants from women with normal BMI showed greater amounts of freshly synthesized ^13^C-DHA-lipids with myo-inositol treatment compared with untreated controls (i.e. myo-inositol response > 0). In contrast, explants from women with higher BMI showed lower amounts of ^13^C-DHA-lipids compared with untreated controls (i.e. myo-inositol response < 0). The intercept from a positive to a negative myo-inositol response occurred around a BMI of 23 kg/m^2^ for all lipids; BMI ≥ 23 kg/m^2^ is the Asian population threshold for being overweight^47^.

**Table 1:** Associations between BMI or maternal glycemia (fasting, 2 h post-load) with ^13^C-DHA lipid in response to myo-inositol treatment (myo-inositol response).

^13^C-lipid	Associations between BMI and myo-inositol response^a^		Associations between fasting glycemia and myo-inositol response^a^		Associations between 2 h glycemia and myo-inositol response^a^	
	Estimate (CI)^b^, unit/(kg/m^2^)	P (BH) value	Estimate (CI)^b^, unit/(mmol/L)	P (BH) value	Estimate (CI)^b^, unit/(mmol/L)	P (BH) value
DG 38:6	− 0.045 (− 0.095,0.005)	0.081	− 0.313 (− 0.851,0.226)	0.238	− 0.088 (− 0.24,0.064)	0.248
DG 40:7	− 0.049 (− 0.105,0.007)	0.083	− 0.499 (− 1.065,0.067)	0.08	− 0.119 (− 0.284,0.046)	0.146
DG 40:8	− 0.043 (− 0.087,0.001)	0.056	− 0.339 (− 0.807,0.128)	0.143	− 0.081 (− 0.216,0.054)	0.218
LPC 22:6	− 0.091 (− 0.167,− 0.016)	0.022	− 0.503 (− 1.37,0.364)	0.238	− 0.13 (− 0.377,0.116)	0.289
LPE 22:6	− 0.098 (− 0.213,0.016)	0.095	− 0.517 (− 1.762,0.728)	0.396	− 0.031 (− 0.391,0.33)	0.872
PC 38:6	− 0.097 (− 0.17,− 0.025)	0.017‡	− 0.29 (− 1.184,0.603)	0.542	0.017 (− 0.239,0.273)	0.887
PE-P 38:6	− 0.095 (− 0.154,− 0.036)	0.004^†‡^	− 0.592 (− 1.313,0.129)	0.107	− 0.175 (− 0.376,0.027)	0.1
PE-P 40:6	− 0.08 (− 0.142,− 0.018)	0.017	− 0.597 (− 1.286,0.092)	0.087	− 0.171 (− 0.365,0.023)	0.09
TG 54:6	− 0.066 (− 0.121,− 0.011)	0.03	− 0.649 (− 1.205,− 0.093)	0.03	− 0.228 (− 0.366,− 0.09)	0.005^§^
TG 54:7	− 0.072 (− 0.148,0.004)	0.07	− 0.738 (− 1.503,0.027)	0.06	− 0.265 (− 0.461,− 0.069)	0.014
TG 56:6	− 0.066 (− 0.119,− 0.013)	0.021^†^	− 0.729 (− 1.233,− 0.225)	0.008^§^	− 0.152 (− 0.313,0.01)	0.076
TG 56:7	− 0.066 (− 0.12,− 0.013)	0.018^†^	− 0.778 (− 1.266,− 0.29)	0.004^§^	− 0.185 (− 0.337,− 0.032)	0.021
TG 56:8	− 0.071 (− 0.121,− 0.02)	0.01	− 0.631 (− 1.172,− 0.091)	0.025	− 0.189 (− 0.337,− 0.04)	0.018
TG 56:9	− 0.071 (− 0.14,− 0.002)	0.054	− 0.821 (− 1.476,− 0.166)	0.019	− 0.243 (− 0.424,− 0.063)	0.016
TG 58:8	− 0.077 (− 0.145,− 0.009)	0.035	− 0.698 (− 1.409,0.013)	0.058	− 0.206 (− 0.404,− 0.008)	0.052
TG 58:9	− 0.074 (− 0.147,0)	0.051	− 0.948 (− 1.616,− 0.279)	0.009^§^	− 0.276 (− 0.461,− 0.091)	0.006
TG 58:10	− 0.065 (− 0.125,− 0.006)	0.034	− 0.832 (− 1.366,− 0.298)	0.005^§^	− 0.226 (− 0.381,− 0.071)	0.007

DG: diacylglycerols, DHA: docosahexaenoic acid, LPC: lyso-phosphatidylcholine, LPE: lyso-phosphatidylethanolamine, PC: phosphatidylcholine, PE: phosphatidylethanolamine, PE-P: phosphatidylethanolamine plasmalogen, TG: triacylglycerol.

^†^Statistically significant after adjusting for fasting glycemia, ^‡^Statistically significant after adjusting for post-load glycemia (2 h), ^§^Statistically significant after adjusting for BMI.

^a^Myo-inositol response represents the relative amount of ^13^C-DHA lipid in placental explants treated with myo-inositol (60 µmol/L) for 48 h compared with control explants from the same placenta not treated with myo-inositol.

^b^Linear regression was run with myo-inositol response as the outcome. The Benjamini-Hochberg (BH) method was used to correct for multiple testing.

When cases were stratified by maternal BMI of 23 kg/m^2^, the amount of most ^13^C-DHA-lipids in the normal BMI population (DG 40:8, PE-P 38:6, PE-P 40:6, seven out of nine TGs) were increased in the myo-inositol treated explants compared with controls (Fig. 1B). In contrast, the amounts of ^13^C-DHA-lipids were generally decreased in the myo-inositol treated explants in the overweight or obese population (Fig. 1B) with significant decreases observed for DG 38:6, and TG 54:6, as well as both the plasmalogens, PE-P 38:6 and PE-P 40:6.

### Association of maternal glycemia with change in amount of placental ^13^C-DHA lipids in response to myo-inositol treatment

**Figure 2:**
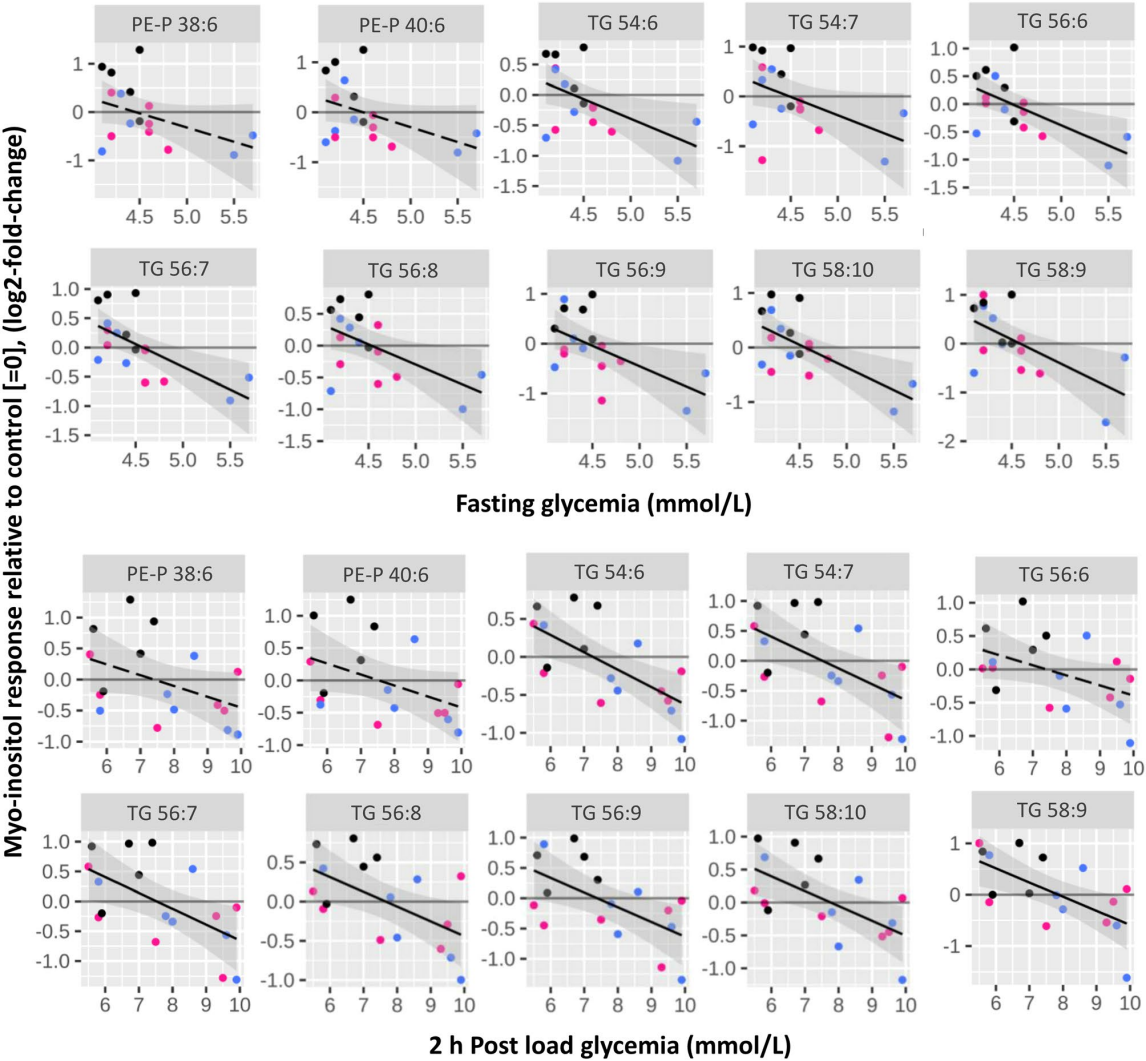
Associations between maternal glycemia (fasting or 2 h post-load) and ^13^C-DHA lipid in response to myo-inositol treatment (myo-inositol response). Myo-inositol response represents the relative amount of ^13^C-DHA lipid in placental explants treated with myo-inositol (60 µmol/L) compared with control explants from the same placenta not treated with myo-inositol. Positive values for myo-inositol response (log2-fold-change) indicate an increase in ^13^C-DHA lipids compared with control (= 0), whilst negative values indicate a decrease. Linear regression was run with myo-inositol response as the outcome and maternal glycemia as the exposure/predictor variable. The Benjamini-Hochberg (BH) method was used to correct for multiple testing. Solid lines show statistically significant associations, while dashed lines show non-significant trends. Shaded areas show 95% confidence intervals. Black: Normal BMI (< 23 kg/m^2^), Blue: overweight (BMI 23 to < 27.5 kg/m^2^), Pink: obese (BMI ≥ 27.5 kg/m^2^).

We had also previously reported that basal placental production of ^13^C-DHA-lipids increases with higher maternal glycemia^12^, and here we examined how myo-inositol treatment in-vitro could alter placental DHA metabolism. With respect to mid-gestation maternal glycemia as a continuum, there was also a negative association between maternal glycemia (both fasting and 2 h post-load) and ^13^C-DHA-TG myo-inositol response (Fig. 2, Table 1). Explants from women with lower glycemia, showed increased amounts of ^13^C-DHA TGs with myo-inositol treatment compared with untreated controls (i.e. myo-inositol response > 0), while decreases were seen in women with higher glycemia (i.e. myo-inositol response < 0). The intercept at which myo-inositol results in no change in myo-inositol response (i.e. at 0) occurred at around 4.4 mmol/L fasting glycemia, which corresponds to the median concentration in the Singapore pregnant population^48^. However, following stratification by GDM status classified by the WHO-2013 criteria that is based on a 3-time point 75 g OGTT^49^, neither the GDM nor the non-GDM group showed significant differences in the amount of ^13^C-DHA-lipids in myo-inositol treated explants compared with controls.

The myo-inositol response of several ^13^C-DHA TG remained significantly negatively associated with both BMI and glycemia, after mutual covariate adjustment of both BMI and glycemia (in the same model) suggesting that both maternal metabolic factors may independently impact TG myo-inositol response (Table 1).

Phospholipid myo-inositol responses were not, however, associated with fasting or 2 h glycemia, either before or after adjusting for BMI (Fig. 2). Furthermore, myo-inositol response for ^13^C-DHA PE-P 38:6 remained significantly negatively associated with BMI after adjusting for maternal fasting glycemia while ^13^C-DHA PC 38:6 and PE-P 38:6 remained significantly negatively associated with BMI after adjusting for maternal post-load glycemia (Table 1). This suggests that phospholipid myo-inositol response is mainly affected by BMI, rather than by maternal glycemia.

### Association of basal ^13^C-DHA processing with change in placental ^13^C-DHA lipids in response to myo-inositol treatment

**Figure 3:**
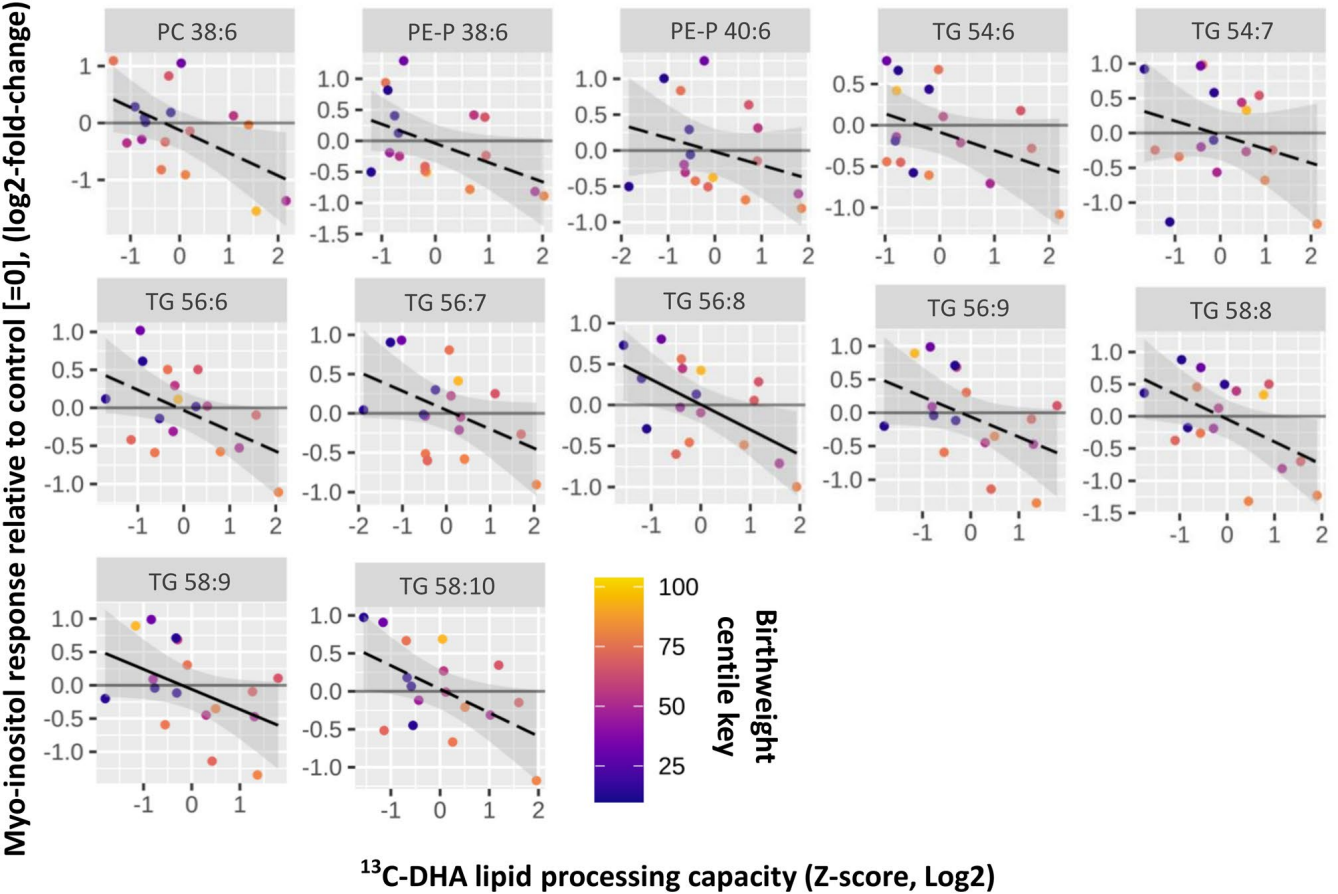
The association of basal lipid processing capacity in control explants (no additional myo-inositol) with myo-inositol response. Myo-inositol response represents the relative amount of ^13^C-DHA lipid in placental explants treated with myo-inositol (60 µmol/L) compared with control explants from the same placenta not treated with myo-inositol. Linear regression was run with myo-inositol response (log2-fold-change) as the outcome and basal lipid processing (calculated by the formula (^13^C-DHA/ (^13^C-DHA + ^12^C-DHA)) Z-score, log2 transformed) as the predictor variable. The Benjamini-Hochberg (BH) method was used to correct for multiple testing. Solid lines show statistically significant associations while dashed lines show non-significant trends. Shaded areas show 95% confidence intervals. Colors indicate birthweight centile.

We had previously found that under control experimental conditions basal ^13^C-DHA lipids in placental explants were positively associated with maternal glycemia and BMI^12^. We therefore investigated if myo-inositol response was dependent upon basal ^13^C-DHA processing capacity [reflected and quantified by the enrichment calculation: ^13^C-DHA/(^13^C-DHA + ^12^C-DHA)]. We found that the higher the basal ^13^C-DHA-lipid processing capacity in control untreated explants, the lower the myo-inositol-induced response of the corresponding lipid (Fig. 3), with significant negative associations observed for TG 56:8 and TG 58:9 (Fig. 3, Table 2). All trend lines crossed the x axis at z-score of 0, demonstrating that myo-inositol treatment decreases the amount of ^13^C-DHA lipids when basal placental ^13^C-DHA processing capacity is above average (as associated with those with higher maternal BMI or glycemia), but increases the amount ^13^C-DHA lipids when basal processing capacity is below average (i.e. as associated with those with normal BMI or lower glycemia). These findings suggest that myo-inositol treatment could have a moderating effect on placental DHA metabolism, directing it towards physiological means.

**Table 2:** Association of basal lipid processing capacity in control explants with myo-inositol response and the association of myo-inositol response with birthweight centile.

^13^C-lipid	(A) Associations between basal lipid processing capacity and myo-inositol response^a^		(B) Associations between birthweight centile and myo-inositol response^a^	
	Estimate (CI), unit/(mmol/L)	P (BH) value	Estimate (CI), unit/(mmol/L)	P (BH) value
DG 38:6	− 0.055 (− 0.309,0.2)	0.964	− 29.127 (− 61.459,3.205)	0.074
DG 40:7	− 0.063 (− 0.347,0.22)	0.722	− 29.09 (− 57.288,− 0.892)	0.044
DG 40:8	− 0.047 (− 0.274,0.18)	0.666	− 30.132 (− 67.089,6.825)	0.103
LPC 22:6	− 0.055 (− 0.466,0.357)	0.982	− 15.748 (− 36.423,4.928)	0.125
LPE 22:6	− 0.203 (− 0.772,0.365)	0.916	− 9.201 (− 24.349,5.947)	0.215
PC 38:6	− 0.4 (− 0.748,− 0.052)	0.054	− 19.848 (− 39.49,− 0.207)	0.048
PE-P 38:6	− 0.31 (− 0.626,0.006)	0.108	− 25.267 (− 46.969,− 3.565)	0.025
PE-P 40:6	− 0.188 (− 0.518,0.142)	0.488	− 23.876 (− 47.143,− 0.61)	0.045
TG 54:6	− 0.224 (− 0.497,0.05)	0.204	− 23.299 (− 51.386,4.789)	0.097
TG 54:7	− 0.202 (− 0.579,0.175)	0.542	− 11.715 (− 34.376,10.946)	0.288
TG 56:6	− 0.27 (− 0.521,− 0.018)	0.074	− 29.832 (− 56.908,− 2.756)	0.033
TG 56:7	− 0.243 (− 0.505,0.019)	0.134	− 31.392 (− 57.644,− 5.14)	0.022
TG 56:8	− 0.308 (− 0.545,− 0.07)	0.028	− 28.624 (− 56.175,− 1.074)	0.043
TG 56:9	− 0.302 (− 0.622,0.019)	0.126	− 18.045 (− 41.682,5.592)	0.125
TG 58:8	− 0.353 (− 0.664,− 0.043)	0.056	− 27.042 (− 47.422,− 6.662)	0.013
TG 58:9	− 0.456 (− 0.747,− 0.164)	0.01	− 22.52 (− 43.147,− 1.892)	0.034
TG 58:10	− 0.311 (− 0.58,− 0.041)	0.054	− 20.887 (− 47.51,5.735)	0.115

DG: diacylglycerols, DHA: docosahexaenoic acid, LPC: lyso-phosphatidylcholine, LPE: lyso-phosphatidylethanolamine, PC: phosphatidylcholine, PE: phosphatidylethanolamine, PE-P: phosphatidylethanolamine plasmalogen, TG: triacylglycerol.

^a^Myo-inositol response represents the relative amount of ^13^C-DHA lipid in placental explants treated with myo-inositol (60 µmol/L) compared with control explants from the same placenta not treated with myo-inositol. (A) Linear regression was run with myo-inositol response (log2-fold-change) as the outcome and basal lipid processing (calculated by the formula (^13^C-DHA/(^13^C-DHA + ^12^C-DHA)) Z-score, log2 transformed) as the predictor variable. Basal lipid processing capacity was measured in control explants with no additional myo-inositol. (B) Linear regression was run with birthweight centile as the outcome and myo-inositol response as the predictor variable. Birthweight centile was standardized for sex and gestational age using local references^65,66^. The Benjamini-Hochberg (BH) method was used to correct for multiple testing.

### Association of change in placental ^13^C-DHA lipids in response to myo-inositol treatment with birthweight centile

**Figure 4:**
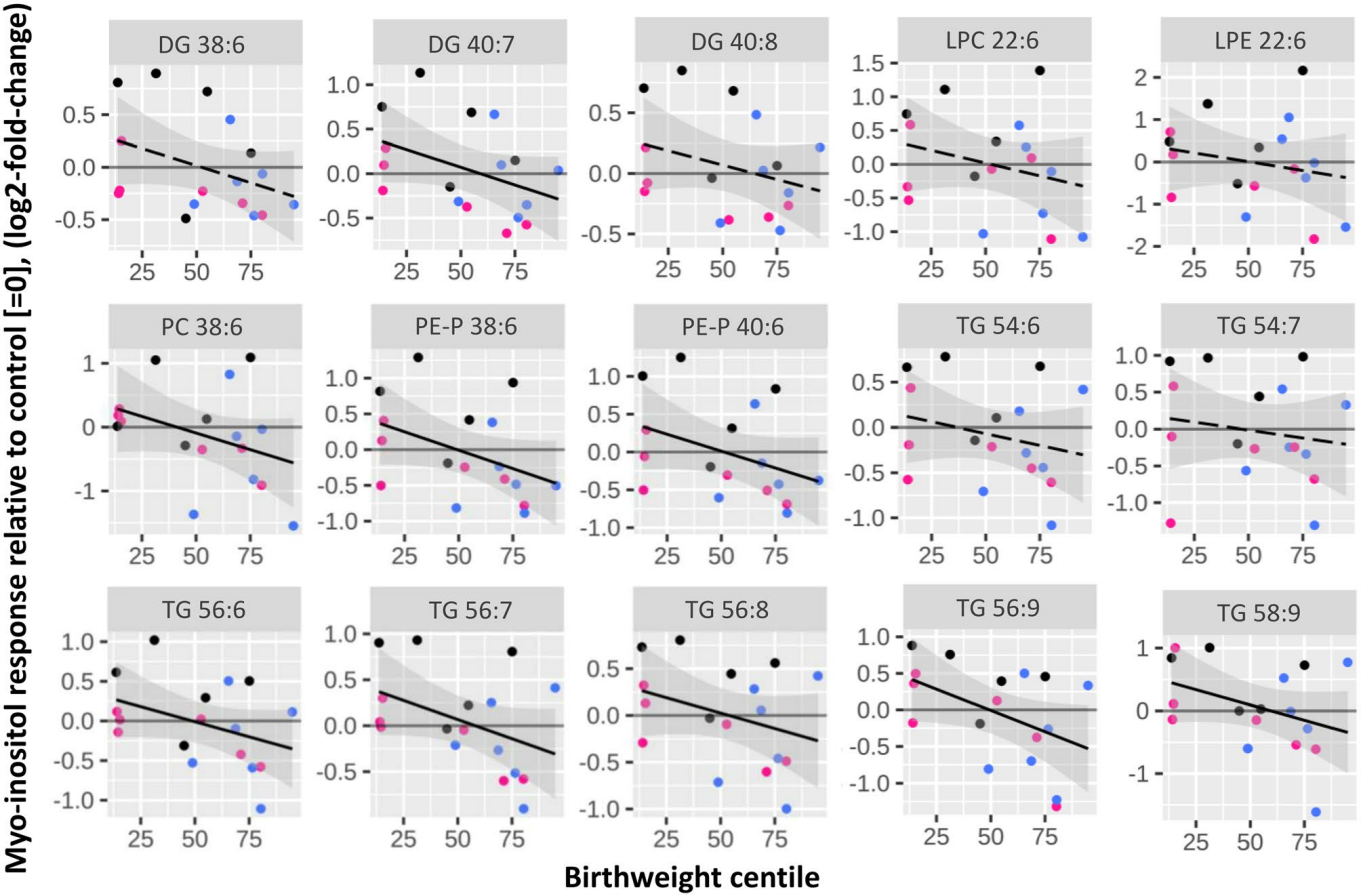
The association of myo-inositol response with birthweight centile. Linear regression was run with birthweight centile as the outcome and myo-inositol response as the predictor variable. Myo-inositol response (log2-fold-change) represents the relative amount of ^13^C-DHA lipid in placental explants treated with myo-inositol (60 µmol/L) compared with control explants from the same placenta not treated with myo-inositol. Birthweight centile was standardized for sex and gestational age using local references^65,66^. The Benjamini-Hochberg (BH) method was used to correct for multiple testing. Solid lines show statistically significant associations while dashed lines show non-significant trends. Shaded areas show 95% confidence intervals. Black: Normal BMI (< 23 kg/m^2^), Blue: overweight (BMI 23 to < 27.5 kg/m^2^), Pink: obese (BMI ≥ 27.5 kg/m^2^).

Next, we examined the associations between myo-inositol induced changes in ^13^C-DHA lipids and birthweight centile (Fig. 4, Table 2). The myo-inositol responses of many ^13^C-DHA lipids were negatively associated with birthweight centile (significant for PC 38:6, PE-P 38:6 and PE-P 40:6, DG 40:7 and five out of nine TGs). Increases in ^13^C-DHA lipids with myo-inositol treatment were observed in placenta supporting babies of lower birthweight centiles where DHA-lipid processing tended to be lower at the basal untreated condition (Figs. 3 and 4). However, decreases in ^13^C-DHA-lipids (i.e. myo-inositol response < 0) were observed in placenta supporting babies of higher birthweight centiles where basal DHA-lipid processing tended to be higher in the untreated condition (Figs. 3 and 4). Intercepts of the axis (i.e. when there is no myo-inositol response, = 0) occurred around the birthweight centile of 50–60 for all DHA-lipids (Fig. 4).

## Discussion

We found that myo-inositol treatment in-vitro decreased incorporation of ^13^C-DHA into placental lipid pools reflected by a decrease in newly synthesized ^13^C-DHA-lipids in placental explants from women with higher BMI or glycemia, where basal placental DHA-processing-capacity is higher. Conversely, myo-inositol increased newly synthesized ^13^C-DHA-lipids in placenta from cases of normal BMI and lower glycemia where basal DHA-lipid processing-capacity is lower. This is consistent with our hypothesis that myo-inositol may act as a moderator of placental ^13^C-DHA lipid metabolism, regulating placental DHA-lipids towards the physiological mean, which may represent the optimum state. As a consequence, myo-inositol may also serve to moderate outcomes which may be influenced by placental ^13^C-DHA lipid metabolism, such as birthweight. This notion is supported by our finding of an increase in placental DHA-lipids in response to myo-inositol treatment in placenta of neonates of lower birthweight centiles, but a decrease at higher centiles.

### Consistency with previous studies

Our previous small (n = 7) study on placental explants from mainly overweight non-GDM women showed that myo-inositol treatment of 30 or 100 µmol/L decreased the amount of the only three ^13^C-DHA-TG lipids detectable using an earlier methodology^45^. Our current study with an improved method can now measure more ^13^C-DHA lipids and included a greater diversity of cases, including women with and without GDM, and with a range of BMIs. We have been able to replicate our earlier findings that myo-inositol decreases ^13^C-DHA-TG when maternal BMI is high, while expanding our results to show that other DHA lipid classes are similarly affected.

Furthermore, a previous study has suggested that the effects of myo-inositol treatment on serum plasmalogens (measured by radiolabeled iodine liquid chromatography assay) in hyperlipidemic human adults, was inversely proportional to serum plasmalogen levels before treatment, with bigger increases seen when serum plasmalogen concentrations were initially lower, and decreases seen when plasmalogen concentrations were initially higher^50^. This phenomenon is consistent with our findings of myo-inositol having a moderating effect on placental DHA metabolism.

### Potential myo-inositol moderation of the effects of BMI and glycemia on placental ^13^C-DHA lipid metabolism

We previously reported that the maternal metabolic factors of BMI and glycemia could program the placenta to increase production of ^13^C-DHA lipids^12^. Myo-inositol appears to have a moderating effect on placental ^13^C-DHA lipid metabolism in-vitro, potentially rectifying the programmed effects of a raised BMI and higher glycemia. In contrast, in cases of lower maternal glycemia and BMI, it might be important to increase placental ^13^C-DHA lipid production to ensure adequate DHA storage reserves for supply of DHA to support growth and development.

Such moderation also translated into the relationship between myo-inositol-responsive placental DHA metabolism with birthweight. Whether BMI and glycemia are simply confounders associated with myo-inositol-responsive placental DHA lipid metabolism and birthweight separately, or whether alterations in placental DHA lipid metabolism (that is amenable to inositol regulation) underlies one of the causal pathways linking maternal BMI and glycemia to birthweight remains unclear. Larger studies and intervention trials will be needed to infer causality and confirm mediation pathways.

### Possible underlying mechanisms of effect

The possible mechanisms involved in how myo-inositol could moderate placental DHA metabolism remains speculative. Since, myo-inositol increases ^13^C-DHA-lipids when maternal BMI and glycemia are low, but decreases ^13^C-DHA-lipids when maternal BMI and glycemia are high, it seems likely that myo-inositol could impact multiple different metabolic pathways, increasing some synthetic processes, but decreasing others, with the net effects dictating the amount of freshly synthesized ^13^C-DHA-lipids in placental explants.

Maternal factors such as BMI may determine the balance of these competing processes, such that subsequent myo-inositol treatment would impact the more predominant of these processes over the others, leading to a change in the overall net effect on each lipid. That myo-inositol treatment affects both TGs and phospholipids in similar directions and to a similar extent suggests that myo-inositol likely has greatest impact upon upstream synthetic processes such as activation into DHA-CoA, rather than downstream processes such as those specific to a particular lipid class or lipid-catabolism.

Likely candidates mediating the myo-inositol effects include acyl-CoA synthetase enzymes, particularly those that are relatively specific to DHA such as ACSL6^51,52^ and a range of acyltransferase enzymes such as glycerol-3-phosphate acyltransferase (GPAT), which was found to be activated by inositol phosphoglycans^53^.

Further, myo-inositol is needed to synthesize phosphatidylinositol (PI) and PI-phosphates (PIP) which are essential signaling molecules in the PI3K-AKT-mTOR pathway, which serves as a major controller of both lipid anabolic and catabolic pathways in many tissues and this pathway is known to be disrupted in obesity as well as diabetes^54–56^. In particular, mTOR is known to regulate PPARγ, which plays a role in regulating placental long chain polyunsaturated fatty acid (LC-PUFA) uptake and metabolism^16,57^. Placental expression of PPARγ mRNA is also positively associated with birthweight^16,57^.

### Myo-inositol moderation of different DHA-lipid classes with increasing maternal BMI and glycemia, and association with birthweight

Changes in placental DHA-lipid metabolism will influence fetal DHA-lipid supply and the availability of DHA-based signaling molecules such as ω-3 series eicosanoids, and hence fetal growth and development. DHA-phospholipid myo-inositol response appears more influenced by BMI than DHA-TG myo-inositol response, with PE-P 38:6 showing the most marked changes. PE-P 38:6 and PE-P 40:6 were also the only lipids to be both significantly increased in the normal BMI group and significantly decreased in the overweight/obese group with myo-inositol treatment. Placental PEs (which includes PE-Ps) are thought to be a major source of un-esterified DHA for the fetus^11^. Also, placental DHA-phospholipids, as well as placental PEs and lyso-phospholipids in general, have previously been linked with birthweight^11,16^. Further, plasmalogens such as PE-Ps, are particularly important for eicosanoid production and also have anti-oxidant properties^58–61^, which influence placental and fetal health, and parturition. It therefore seems likely that myo-inositol-induced changes in DHA-phospholipid metabolism, particularly PE-P metabolism, could impact maternal-placental-fetal metabolism, and hence fetal growth and development^58–61^. Thus, myo-inositol interventions could be particularly pertinent in maternal obesity.

In contrast, DHA-TG myo-inositol response appears more influenced by glycemia, with stronger effects observed for TGs containing more poly-unsaturated fatty acids (e.g. TG 58:9, 58:10), than those containing more saturated fatty acids (e.g. TG 54:6, TG 54:7, TG 56:6). Since the basal placental processing capacity of ^13^C-DHA TG is markedly associated with birthweight, myo-inositol moderation of DHA-TGs could also plausibly have appreciable impact on birthweight.

### Observations of myo-inositol influences on birthweight in clinical studies

In a meta-analysis of three clinical trials of antenatal myo-inositol supplementation in Italian women at risk of GDM, birthweight and macrosomia risk was reduced^62^. A recent clinical trial conducted in women diagnosed with GDM, also showed that supplementation with myo-inositol with α-lactalbumin reduced fetal abdominal circumference and adiposity^63^. In addition, the GUSTO mother–offspring cohort study showed that high placental inositol was associated with the attenuation of fasting glycemia-associated increase in birthweight and neonatal adiposity^34^, consistent with the notion of placental inositol acting to moderate the fetal growth-promoting effects of maternal glycemia. Our present findings suggest that inositol moderation of placental DHA-lipid metabolism may constitute part of the underlying mechanism of effect. With a lower placental inositol content in GDM^33^, lack of such regulatory moderation of changes in placental DHA-lipid metabolism may increase susceptibility to disorders of fetal growth^64^.

These studies and ours raise a novel postulation that myo-inositol supplementation, possibly in part through modulation of placental DHA metabolism*,* may contribute to the moderation of fetal growth, bringing birthweight towards the physiological mean, from both sides of the maternal BMI and glycemia spectrum. Since our study did not include growth-restricted pregnancies, it is unknown if myo-inositol could also be associated with moderation of birthweight at the lower extreme of the growth spectrum.

Our current work is also in keeping with the hypothesis that the fetus exports myo-inositol to the placenta as a signal to tailor its nutrient supply according to fetal demand^31^. Overall placental myo-inositol content is also believed to come from placental endogenous production and from the maternal compartment, hence, the potential for maternal myo-inositol supplementation to have an impact.

### Strengths and limitations of study

Our novel ^13^C-DHA in-vitro methods are a major study strength as they enabled us to measure the lipid synthetic and metabolic capacity of placenta, without confounding by maternal/fetal DHA metabolism and prevailing DHA supply to placenta. However, such methods are not easily scaled to large sample sizes, and placental lipid metabolism can only be assessed following delivery. The modest sample size of this in-vitro study thus precludes more complex analyses to confirm if myo-inositol effects on placental DHA-lipid metabolism could indeed moderate the effects of maternal BMI and glycemia on birthweight. Thus, replication in larger cohorts is required to confirm these findings. The participants of this study were Chinese and Indian, limiting the generalizability of these findings to other populations. A balance of GDM/non-GDM and high/normal BMI cases were selected and the measurements are therefore not representative of normal variation within the general pregnant population. We also have not studied how changes in placental explant DHA-lipids relate to DHA-lipids in maternal and fetal/cord circulation. The incorporation of ^13^C-DHA into other lipids not discussed in this paper could not be reliably quantified using this methodology. We also note that placental DHA metabolism is only a small part of overall lipid metabolism, and the placental lipid metabolism of other fatty acids may react differently to inositol^45^. Further, DHA was studied in isolation, but in-vivo the presence of many other fatty acids may influence placental DHA metabolism. An understanding of the combined effects of all placental lipid metabolism on fetal growth and development, and how this may be modulated by inositol will be required in order to fully understand the role of myo-inositol in placental lipid metabolism, and its impact on fetal size.

## Conclusion

Treatment of placental explants with myo-inositol moderated the amount of freshly synthesized ^13^C-DHA lipids. The direction of effect depended strongly on maternal BMI and glycemia, with myo-inositol bringing placental DHA metabolism towards the physiological mean. This raises the novel postulation that myo-inositol intervention could help to moderate placental ^13^C-DHA lipid metabolism and rectify transplacental DHA supply in pathological conditions, and potentially improve dysregulated DHA-dependent processes such as disordered fetal growth. Further research is needed to confirm this postulation.

## Methods

### Recruitment and clinical characteristics of participants

Placenta (n = 17) were collected from women recruited at the National University Hospital, Singapore with informed written consent between 2018 to 2020. Ethical approval was obtained from the National Healthcare Group Domain Specific Review Board (2016/00183). All methods were performed in accordance with the relevant guidelines and regulations. Universal screening for gestational diabetes (GDM) was performed at mid-gestation by a three time-point 75 g oral glucose tolerance test (OGTT) using WHO 2013 criteria^49^. Women with pre-existing type 1 or 2 diabetes mellitus were excluded. Non-smoking mothers who delivered singletons after 37 weeks’ gestation by elective Cesarean section, with newborns who were greater than the 10th percentile (by local sex- and gestational age-standardized references^65,66^) were eligible. Nine GDM and eight non-GDM cases were recruited and matched for first trimester BMI (similar mean (SD) kg/m^2^: GDM: 24.1 (3.9); non-GDM: 26.6 (5.0)), hence neither fasting glycemia nor post-load glycemia were significantly associated with BMI. Scientists running explant experiments were blinded to the clinical characteristics of the women and neonates involved until the completion of all explant culture experiments and LCMS. Table 3 shows the characteristics of the study population.

**Table 3:** Clinical characteristics of study population.

**Maternal characteristics**	Mean (SD) or n (%)
Maternal age (years)	33.4 (2.9)
Chinese:Indian ethnicity	65%:35%
Maternal BMI in first trimester (kg/m^2^)	25.4 (4.6)
Fasting glycemia (mmol/L)	4.5 (0.5)
1 h glycemia (mmol/L)^a^	9.0 (1.7)
2 h glycemia (mmol/L)^a^	7.6 (1.6)
**Neonatal characteristics**	Mean (SD) or n (%)
Gestational age at birth (days)	271.4 (5.9)
Female neonates	52%
Birthweight (g)	3276 (323)
Birthweight centile (%)^b^	61 (30)

^a^In mid-gestation following 75 g OGTT.

^b^Standardized for sex and gestational age using local references^65,66^.

### Placental collection and placental tissue culture

Villous explants were prepared and cultured as previously described^12^. Briefly, multiple random biopsies of villous explants (approximately 3 × 3 × 3 mm) from each placenta were cultured in each well of 12-well plates in serum-free CMRL media (1.8 mL, contain 0.3 µmol/L inositol) with 1.5% BSA with either no DHA or ^13^C_22_-DHA (24 µmol/L, 99 atom % ^13^C, 99% CP, Cambridge isotope laboratories). Paired samples from each placenta were cultured with either no additional myo-inositol or 60 µmol/L myo-inositol (Sigma, > 99% pure, Saint Louis, MO), each condition in triplicate. The normal physiological circulating concentration of maternal myo-inositol is 20–50 µmol/L, while umbilical cord blood myo-inositol ranges between 45 and 125 µmol/L^67,68^, so 60 µmol/L was added to determine the effects of myo-inositol, similar to a supplemented state^68^.

### Lipid extractions and quantification by LCMS

^13^C-DHA-lipids were extracted and analyzed as previously described^12^. The frozen placental explants were freeze-dried, weighed, and then lysed in phosphate buffered saline (PBS, 1200 μL) using a bead-ruptor homogenizer. Lysate (40 µL) was mixed with 800 µL Butanol/Methanol (1:1) and 10 µl internal standard mix, samples vortexed, then sonicated for 30 min in an ice bath, then shaken for 30 min at 4 °C. Samples were then centrifuged at 13,000 rpm for 10 min and the supernatant stored at − 80 °C. Lipid extracts (5 μL) were injected into an Agilent 6490 triple quadrupole (QQQ) liquid chromatography mass spectrometry (LC–MS/MS) instrument and analyzed using a targeted dynamic multiple reaction monitoring (dMRM) method containing transitions for individual ^13^C_22_-DHA-lipids and their co-eluting ^12^C_22_-DHA-containing partners^12^. The LCMS quantification method was previously developed^12^ based on the principle that ^13^C-DHA-lipids have identical physiological and chromatographic properties to ^12^C-DHA-lipids, but their mass will be 22 Daltons more than their endogenous counterparts. This enabled hypothetical ^13^C-DHA-lipid dMRM transitions to be calculated for TGs, DGs and phospholipids that could possibly contain DHA, based on the lipidomic methods of the Baker Institute^12^. Transitions were kept if ^13^C-DHA-lipid peaks exactly co-eluted with their ^12^C-DHA counterparts and were absent in placental samples not incubated with ^13^C-DHA^12^.

### Data analysis

The amount of ^13^C-DHA-lipids in placental explants was calculated using internal standards as previously described^12^. The effects of myo-inositol addition were expressed as fold-changes in the amount of labeled lipid relative to control tissue (with no additional myo-inositol) from the same placenta. The fold-change was then log-2-transformed to give the relative response to myo-inositol (i.e. myo-inositol response, log2-fold-change). A myo-inositol response > 0 indicates an increase in ^13^C-DHA lipids compared to the control, while a response < 0 indicates a decrease.

Linear regression was used to assess the myo-inositol response of each lipid with each variable of interest (predictor) namely maternal BMI, fasting glycemia, post-load 2 h glycemia, maternal age, or gestational age at delivery. Where indicated, multiple linear regression was then performed with mutual adjustments for these variables. Next, linear regression was conducted for birthweight centile (outcome) with the myo-inositol response of each lipid (one lipid per model) as predictor. The Benjamini-Hochberg method was used to correct for multiple testing to minimize false discovery. Repeated measures analysis of variance was used to test whether myo-inositol treatment altered the relative amount of placental ^13^C-DHA lipid in placental explants treated with myo-inositol compared with the control and the effects of covariate correction (one lipid per model). Statistical significance was set at a two-sided alpha level of *p* < 0.05. Data was analyzed in R-studio (Double Marigold, 2020) using *R* version "Kick Things" with tidyverse^69^ and tidymodels^70^ packages. Graphs were made using the ggplot2^71^ and viridis packages^72^.

### Informed consent statement

Placenta were collected with informed written consent.

## Data Availability

Some or all datasets generated during and/or analyzed during the current study are not publicly available but are available from the corresponding author on reasonable request.

## Abbreviations

BMI: Body mass index
BSA: Bovine serum albumin
CMRL: Connaught Medical Research Laboratories
DG: Diacylglycerols
DHA: Docosahexaenoic acid
dMRM: Dynamic multiple reaction monitoring
GDM: Gestational diabetes mellitus
IS: Internal standard
LC–MS/MS: Tandem liquid chromatography mass spectrometry
LC-PUFA: Long-chain polyunsaturated fatty acid
LPC: Lyso-phosphatidylcholine
LPE: Lyso-phosphatidylethanolamine
PC: Phosphatidylcholine
PE: Phosphatidylethanolamine
PE-P: Phosphatidylethanolamine plasmalogen
PUFA: Polyunsaturated fatty acids
TG: Triacylglycerol
